# A multi PDZ-domain protein Pdzd2 contributes to functional expression of sensory neuron-specific sodium channel Na_V_1.8

**DOI:** 10.1016/j.mcn.2009.07.003

**Published:** 2009-10

**Authors:** Dongmin Shao, Mark D. Baker, Bjarke Abrahamsen, Francois Rugiero, Misbah Malik-Hall, W.-Y. Louisa Poon, Kathryn S.E. Cheah, Kwok-Ming Yao, John N. Wood, Kenji Okuse

**Affiliations:** a Division of Cell and Molecular Biology, Faculty of Natural Sciences, Imperial College London, London, UK; b London Pain Consortium, UK; c Department of Biology, University College London, London, UK; d Institute of Cell and Molecular Science, Queen Mary University of London, London, UK; e Department of Biochemistry and Centre for Reproduction, Development and Growth, LKS Faculty of Medicine, University of Hong Kong, HK, China

## Abstract

The voltage-gated sodium channel Na_V_1.8 is expressed exclusively in nociceptive sensory neurons and plays an important role in pain pathways. Na_V_1.8 cannot be functionally expressed in non-neuronal cells even in the presence of β-subunits. We have previously identified Pdzd2, a multi PDZ-domain protein, as a potential interactor for Na_V_1.8. Here we report that Pdzd2 binds directly to the intracellular loops of Na_V_1.8 and Na_V_1.7. The endogenous Na_V_1.8 current in sensory neurons is inhibited by antisense- and siRNA-mediated downregulation of Pdzd2. However, no marked change in pain behaviours is observed in Pdzd2-decificent mice. This may be due to compensatory upregulation of p11, another regulatory factor for Na_V_1.8, in dorsal root ganglia of Pdzd2-deficient mice. These findings reveal that Pdzd2 and p11 play collaborative roles in regulation of Na_V_1.8 expression in sensory neurons.

## Introduction

Voltage-gated sodium channels confer excitability on neurons. The sensory neuron-specific tetrodotoxin (TTX)-resistant sodium channel Na_V_1.8 is shown to have a specific role in the detection of noxious thermal, mechanical and inflammatory stimuli (Akopian et al., 1999; Okuse, 2007). Voltage-gated sodium channel comprise an α-subunit and accessory β-subunits. The β-subunits promote functional channel expression and modulate the biophysical properties of the channels (Isom, 2000). Unlike other voltage-gated sodium channels, introduction of Na_V_1.8 cDNA into heterologous cells such as COS-7 and CHO cells does not result in functional channel expression (England et al., 1998), and co-expression of accessory β-subunits does not help the functional Na_V_1.8 expression (Baker et al., 2004). This suggests that Na_V_1.8 requires other regulatory proteins for its functional expression.

Using a yeast two-hybrid screening, we identified p11, the light chain of annexin II, as a novel regulatory factor for Na_V_1.8 (Okuse et al., 2002). p11 binds directly to the N-terminus of Na_V_1.8 (Poon et al., 2004) and promotes the translocation of Na_V_1.8 to the plasma membrane resulting in functional channels. Although p11 facilitates the functional expression of Na_V_1.8 in CHO cells, the expressed current is considerably smaller (100–150 pA) compared to the endogenous Na_V_1.8 current in sensory neurons (> 3 nA) (Akopian et al., 1999) and other sodium channels expressed in CHO cells (> 3 nA, Na_V_1.2, Ragsdale et al., 1991; > 4 nA, Na_V_1.3, Meadows et al., 2002). The activation of Na_V_1.8 currents observed in CHO cells transfected with p11 cDNA is considerably more depolarised than the endogenous currents observed in dorsal root ganglia (DRG) neurons (Okuse et al., 2002). This suggests that there may be yet another permissive factor to promote functional expression of Na_V_1.8 in sensory neurons.

Here we report identification of Pdzd2 (PDZ domain containing 2), a widely expressed multi-PDZ (PSD-95/Dlg-A/ZO-1)-domain protein also known as Papin and PDZK3, as a channel partner that binds directly to Na_V_1.8 and plays an essential role in functional expression of the channel. We found that the loss of Pdzd2 in Pdzd2-deficient mice is compensated by upregulation of p11 mRNA, potentially masking the lack of analgesic phenotype in Pdzd2-deficient mice. Pdzd2 and p11, therefore, act in concert to control functional Na_V_1.8 expression in DRG neurons.

## Results

### Pdzd2 is expressed in both large and small diameter sensory neurons

**Fig. 1:**
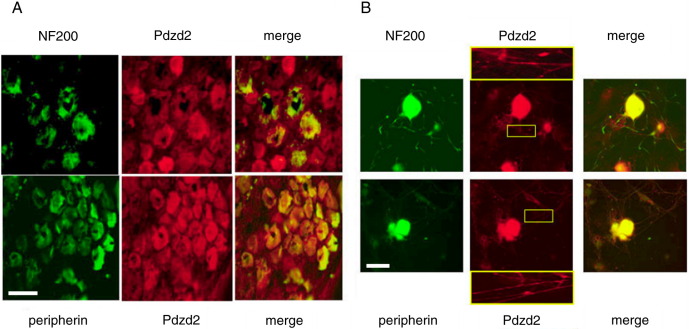
Pdzd2 is expressed in both large and small diameter sensory neurons in DRG. (A) Rat DRG sections (A) and cultured rat DRG neurons (B) stained with anti-NF200, peripherin, and Pdzd2 antibodies. Pdzd2-like immunoreactivity was detected in both large and small diameter sensory neurons, and localised in both cell bodies and neurites. Insets show distribution of Pdzd2 throughout the neuronal fibres of DRG neurons. Scale bars are 50 μm.

Immunohistochemistry was performed on a section of rat DRG. Pdzd2-like immunoreactivity was detected in both NF200 positive large diameter and peripherin positive small diameter neurons in DRG (Fig. 1A). This is consistent with the expression of Pdzd2 mRNA in DRG (Malik-Hall et al., 2003). As majority of peripherin positive DRG neurons express Pdzd2; it is likely that Na_V_1.8 is co-expressed with Pdzd2 in these cells. Expression of Pdzd2 was also examined in cultured DRG neurons. Pdzd2-like immunoreactivity was localised in both cell bodies and neurites in cultured DRG neurons (Fig. 1B).

### Pdzd2 binds to Na_V_1.8 and Na_V_1.7

**Fig. 2:**
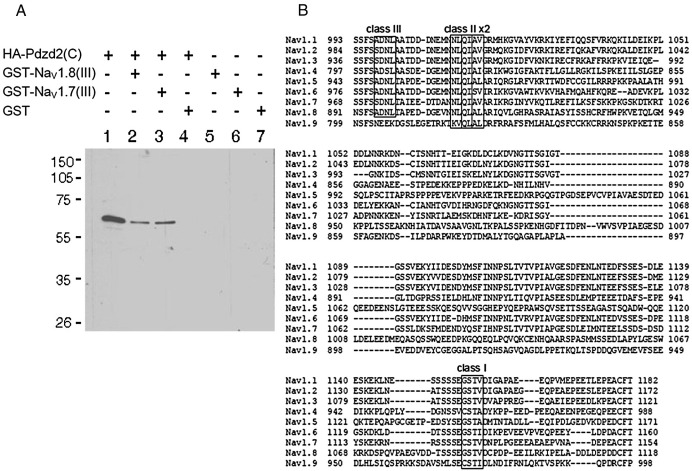
Pdzd2 binds to the intracellular loops between domains 2 and 3 of Na_V_1.8 and Na_V_1.7. (A) HA-tagged rat Pdzd2 C-terminal fragment, HA-Pdzd2(C), showed direct and specific association with the intracellular loops between domains 2 and 3 of rat Na_V_1.8 (lane 2) and Na_V_1.7 (lane 3) in GST pull-down assay. Lane 1 serves as a positive control for HA-Pdzd2(C) on SDS-PAGE. (B) Sequence alignment of the intracellular loops between domains 2 and 3 of the rat voltage-gated sodium channels. Conserved canonical PDZ-binding motifs designated classes I, II, and III are shown in rectangle.

To examine the binding of Pdzd2 to Na_V_1.8 *in vitro*, we performed GST pull-down assays using the haemagglutinin (HA)-tagged rat Pdzd2 C-terminal fragment (201aa), HA-Pdzd2(C), and the intracellular loop between domains 2 and 3 of rat Na_V_1.8 (amino acid positions 893 to 1148) fused to GST (glutathione S-transferase), GST-Na_V_1.8(III). Fig. 2A shows successful pull-down of HA-Pdzd2(C) by GST-Na_V_1.8(III) (lane 2). Purified GST (Fig. 2A, lane 4) failed to pull-down HA-Pdzd2(C), demonstrating that HA-Pdzd2(C) binds specifically to the intracellular loop between domains 2 and 3 of Na_V_1.8. As recent findings revealed an essential role of Na_V_1.7 in nociception (Nassar et al., 2004; Cox et al., 2006; Fertleman et al., 2006; Lee et al., 2007; Zhang et al., 2007), we tested the binding ability of Pdzd2 to Na_V_1.7. The intracellular loop between domains 2 and 3 of rat Na_V_1.7 pulled down HA-Pdzd2(C) efficiently (Fig. 2A, lane 3). The amino acid sequence of the intracellular loop of voltage-gated sodium channels revealed conserved PDZ-binding motifs (Fig. 2B). These PDZ-binding motifs are also conserved in other species (mouse and human, data not shown). This suggests Pdzd2 may bind to the wide range of voltage-gated sodium channels.

### Pdzd2 does not help translocation of Na_V_1.8 to the plasma membrane in CHO cells

**Fig. 3:**
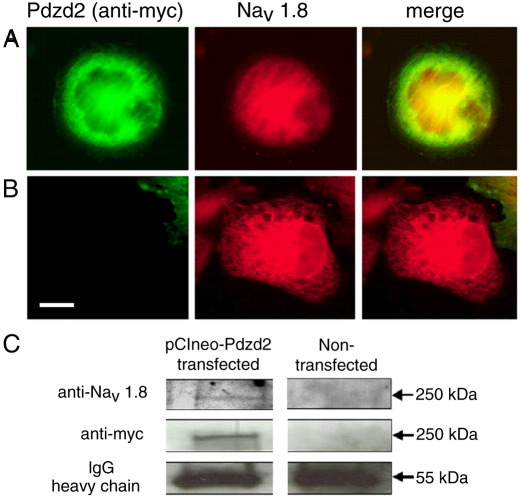
Pdzd2 does not help the translocation of Na_V_1.8 to the plasma membrane in CHO cells. (A) Transiently expressed full length rat Pdzd2 (visualised by anti-myc antibody) did not show specific localisation in the plasma membrane in CHO-SNS22 cells. The expression of Pdzd2 also did not cause translocation of Na_V_1.8 to the plasma membrane. The scale bar indicates 5 μm. (B) The localisation of Na_V_1.8 in Pdzd2 transfected cells was clearly different from non-transfected cells. (C) Na_V_1.8 was co-immunoprecipitated with myc-tagged Pdzd2 from the Pdzd2 transfected CHO-SNS22 cells by anti-myc antibody.

We have previously reported that p11 binds to the amino terminus of Na_V_1.8 and promotes the translocation of Na_V_1.8 to the plasma membrane in CHO-SNS22, a CHO cell line stably transfected with rat Na_V_1.8 cDNA that expresses cytosolic Na_V_1.8 (Okuse et al., 2002). In order to test if Pdzd2 helps translocation of Na_V_1.8 to the plasma membrane, we transfected CHO-SNS22 cells with a myc-tagged full length Pdzd2 cDNA. The myc-Pdzd2 expressed in CHO-SNS22 cells did not show specific localisation in the plasma membrane (Fig. 3A) unlike in MDCK cells (Ohno et al., 2002). The expression of myc-Pdzd2 also did not help translocation of Na_V_1.8 into the plasma membrane in CHO-SNS22 cells (Fig. 3A). The localisation of Na_V_1.8 in myc-Pdzd2 transfected cells was different from non-transfected cells which showed more distinct staining in organelle (Fig. 3B). The interaction between Na_V_1.8 and Pdzd2 in the myc-Pdzd2 transfected CHO-SNS22 cells was confirmed by co-immunoprecipitation (Fig. 3C).

### Pdzd2 is essential for functional expression of Na_V_1.8 current in DRG neurons

**Fig. 4:**
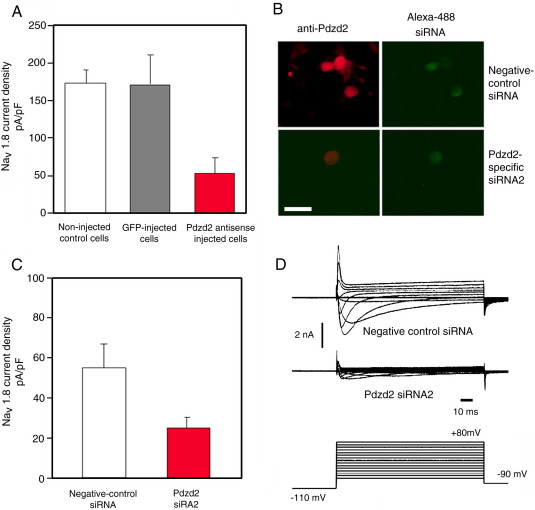
Pdzd2 is required for expression of Na_V_1.8 currents in rat DRG neurons. (A) Pdzd2 antisense mRNA expression in DRG neurons caused a loss of Na_V_1.8 current density. The Pdzd2 antisense expression vector, pcDNA3-AS(Pdzd2), was microinjected into the nuclei of cultured DRG neurons and the TTX-resistant Na^+^ current density was measured 2 days later. (B) Transfection of Pdzd2-specific siRNA (Pdzd2 siRNA2) into cultured DRG neurons caused efficient and specific down-regulation of endogenous Pdzd2 protein expression. The scale bar indicates 50 μm. (C) The transfection of Pdzd2 siRNA2 caused significant reduction of the Na_V_1.8 current density in DRG neurons. The TTX-resistant Na^+^ current density was measured 3 days after transfection of siRNA. (D) Example TTX-resistant inward current traces from negative control siRNA and Pdzd2 siRNA2 treated DRG neurons.

Pdzd2 has been shown to localise in the plasma membrane of epithelial cells with Erbin which has been implicated in trafficking of Erb-B2 and the polarity of epithelial cells (Ohno et al., 2002). We tested the possible regulatory role of Pdzd2 on Na_v_1.8 in sensory neurons by microinjection of the Pdzd2 antisense expression vector, pcDNA3-AS(Pdzd2), with a GFP expression vector pRK7-GFP into the nuclei of cultured DRG neurons. The introduction of pcDNA3-AS(Pdzd2) caused a great loss (71%) of the mean peak Na_V_1.8 current density compared with control neurons injected with pRK7-GFP only (Fig. 4A). We also employed chemically synthesised small interference RNA (siRNA). Transfection of Pdzd2-specific siRNA (Pdzd2 siRNA2) into cultured DRG neurons caused distinct down-regulation of Pdzd2 (Fig. 4B). Negative control siRNA which has no homology to any known mammalian gene failed to downregulate the Pdzd2 expression despite its high transfection efficiency (> 50%), suggesting that Pdzd2 siRNA2 effectively and specifically downregulates Pdzd2 expression in cultured DRG neurons. The transfection of Pdzd2 siRNA2 also reduced more than 50% of the mean peak Na_V_1.8 current density in DRG neurons compared with the neurons transfected with the negative control siRNA (Fig. 4C). Examples of TTX-resistant inward currents recorded from negative control and Pdzd2 siRNA2 treated rat DRG neurons are shown in Fig. 4D.

### Pdzd2-deficient mice show no marked changes in pain behaviour

**Fig. 5:**
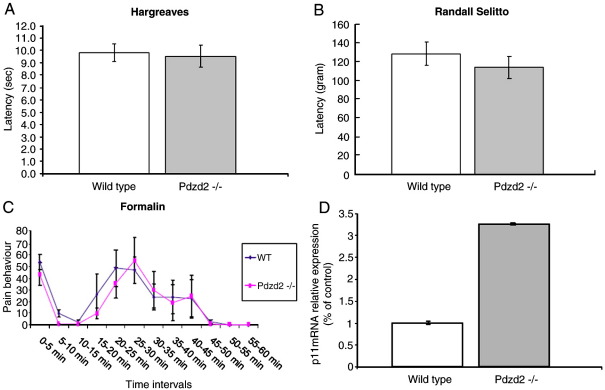
Responses to acute pain and chronic inflammatory pain stimulations are normal in Pdzd2-decificent mice. (A) Noxious radiant heat stimulation using Hargreaves' apparatus. (B) Noxious mechanical pressure applied to the tail using the Randall–Selitto apparatus. (C) Inflammatory pain behaviour after intraplantar injection of 20 μl of 5% formalin. (D) p11 mRNA expression in DRG of wild type and Pdzd2-deficient mice. Real-time PCR data shows that the p11 mRNA levels are significantly increased in Pdzd2-deficient mice as compared with wild type animals (3.3 fold, *p* = 0.03, *t* test). Data shown are normalised to cyclophilin-A and quantitated based on a standard curve.

In order to test acute pain behaviour in Pdzd2-deficient mice, Hargreaves' test (noxious radiant heat) and the Randall–Selitto apparatus (mechanical pressure) were used. There was no difference in paw withdrawal latency in response to noxious radiant heat between Pdzd2-deficient mice and wild type animals (Fig. 5A). Despite significant increases in pain thresholds to noxious mechanical stimuli applied by the Randall–Selitto apparatus that were observed in Na_V_1.8 knockout (Akopian et al., 1999) and p11 knockout mice (Foulkes et al., 2006), there was no marked difference between Pdzd2-deficient mice and wild type animals (Fig. 5B). Intraplantar injection of formalin was used as a model of chronic inflammatory pain. A typical biphasic response in both Pdzd2-deficient and wild type mice was observed. Although there was a slightly delayed reaction of the second phase of the formalin test in Pdzd2-deficient mice, the overall pain behaviour was not significantly different between Pdzd2-deficient and wild type mice (Fig. 5C). This is consistent with the findings in Na_V_1.8 knockout (Nassar et al., 2005) and p11 knockout mice (Foulkes et al., 2006).

### p11 is compensatorily upregulated in Pdzd2-deficient mice

**Fig. 6:**
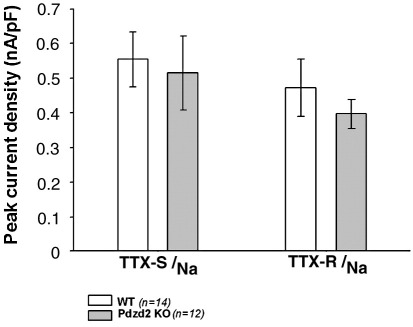
TTX-sensitive and resistant Na^+^ currents in cultured DRG neurons of wild type and Pdzd2-deficient mice. Both TTX-resistant and sensitive Na^+^ current densities were not significantly different between wild type and Pdzd2-deficient mice. TTX-sensitive peak Na^+^ current densities were obtained by subtracting peak TTX-resistant Na^+^ currents from total peak Na^+^ currents.

Sodium currents were recorded from small diameter DRG neurons of wild type and Pdzd2-deficient mice. The TTX-resistant current densities which represent Na_V_1.8 were not significantly different between wild type and Pdzd2-deficient mice (Fig. 6). Thus the lack of analgesic phenotype in Pdzd2-deficient mice is due to the unchanged functional Na_V_1.8 expression on the plasma membrane of DRG neurons. As we reported previously, p11 binds to Na_V_1.8 and promotes its translocation to the plasma membrane, producing functional channels (Okuse et al., 2002). The normal expression of TTX-resistant current in Pdzd2-deficient mice may reflect developmental compensatory upregulation of p11 which could mask the loss of Pdzd2 and help maintain the functional Na_V_1.8 expression in DRG neurons. We examined p11 mRNA levels in DRG of wild type and Pdzd2-deficient mice using quantitative real-time RT-PCR. The wild type mice reactions reached the threshold cycle (Ct) at 26.46 ± 0.67 whereas the Pdzd2-deficient mice reactions reached Ct at 24.67 ± 0.09 cycles. The calculated copy numbers of p11mRNA were 7329 ± 187 (wild type) and 23892 ± 89 (Pdzd2-deficient mice) respectively, thus the expression of p11 mRNA in DRG was significantly (3.3-fold) increased in Pdzd2-deficient mice as compared with wild type animals (Fig. 5D).

## Discussion

In the present study, we show that Pdzd2 binds directly to the intracellular loop between domains 2 and 3 of Na_V_1.8. We also show that the endogenous Na_V_1.8 current in sensory neurons is inhibited specifically by antisense and siRNA-mediated downregulation of Pdzd2. These observations suggest that the interaction between Pdzd2 and Na_V_1.8 is essential for the functional expression of the channel. Although binding of PDZ domains to internal sites in target proteins is not common, there have been a few examples of internal PDZ-binding sites reported (Shieh and Zhu, 1996; Gee et al., 1998). It is also possible that Pdzd2 binds the sodium channels via non-canonical sequence defined by their structural features (Harris and Lim 2001).

In contrast to p11 which promotes the translocation of Na_V_1.8 to the plasma membrane in CHO cells (Okuse et al., 2002), Pdzd2 does not help the surface expression of Na_V_1.8 in CHO cells which suggests that Pdzd2 is a necessary but insufficient factor for Na_V_1.8 expression. This may be explained by lack of other regulatory factors responsible for Pdzd2-mediated membrane translocation of Na_V_1.8. It is also plausible that Pdzd2 is required for stabilising Na_V_1.8 in the plasma membrane as a scaffolding protein rather than trafficking of Na_V_1.8.

The C-terminal fragment (201aa) of Pdzd2 is sufficient for binding to the intracellular loop of Na_V_1.8. Unlike p11 which binds exclusively to Na_V_1.8 among the voltage-gated sodium channels (Poon et al., 2004), Pdzd2 also binds to Na_V_1.7. Na_V_1.7 is known to be expressed in all types of DRG neurons including large diameter neurons (Sangameswaran et al., 1997). It is therefore plausible that Pdzd2 in large diameter sensory neurons may play an important role in nociception by binding to Na_V_1.7. The C-terminal fragment of Pdzd2 possesses two of six PDZ domains found in full length Pdzd2. Pdzd2 is also shown to associate with δ-catenin/NPRAP and p0071 via the second PDZ domain (Deguchi et al., 2002), and with GABA_B_R2 receptor via the first PDZ domain (Balasubramanian et al., 2007). Mutation of the PDZ-binding motif in GABA_B_R2 results in decrease of surface-expressed GABA_B_R2, indicating the stability and signalling of GABA_B_ receptor is modulated via interactions with Pdzd2. The surface expression of Na_V_1.8 in sensory neurons may be regulated by similar machinery involving Pdzd2 and its other binding partners through its remaining PDZ domains.

There is no marked difference in pain threshold to acute noxious radiant heat stimulus between the Pdzd2-deficient and wild type mice. This is consistent with the findings in the p11 knockout mice (Foulkes et al., 2006), but not with the Na_V_1.8 and Na_V_1.7 null mutants where paw withdrawal latencies following exposure to a noxious radiant heat stimulus are increased compared with control animals (Akopian et al., 1999; Nassar et al., 2004). Response to the noxious pressure is also not altered in the Pdzd2-deficient mice, inconsistent with the data observed in the Na_V_1.8 knockouts and the p11 and Na_V_1.7 knockout mice. These contradictions may be explained by the significant compensatory upregulation of p11mRNA in DRG of the Pdzd2-deficient mice, as the fact that p11 promotes the functional expression of Na_V_1.8 into the plasma membrane of sensory neurons. It is also plausible that Na_V_1.8 itself is compensatorily upregulated in the Pdzd2-deficient mice, because Pdzd2 starts to be expressed earlier (E10.5, K. Cheah & K. Yao, in preparation) than Na_V_1.8 which can be visible at E13 the earliest (Akopian et al., 1996). There may also be yet to be identified Pdzd2-related PDZ proteins which could compensate the loss of Pdzd2. It is, therefore, important to study the involvement of Pdzd2 in nociceptor function using a system without developmental compensatory effects such as inducible knockout mice or siRNA injection to the animals.

## Experimental methods

### Immunohistochemistry

Frozen rat DRG sections (10 μm thick), cultured DRG neurons, and CHO-SNS22 cells were fixed for 15 min in 4% paraformaldehyde on ice and subsequently incubated with specific antibodies. The following antibodies were used; mouse anti-Pdzd2 (Yeung et al., 2003), rabbit anti-Na_V_1.8 (Okuse et al., 2002), rabbit anti-NF200, rabbit anti-peripherin (Chemicon), mouse anti-myc, Alexa Fluor 488 goat anti-mouse IgG, Alexa Fluor 488 goat anti-rabbit IgG, Rhodamine Red goat anti-rabbit IgG, and Rhodamine Red goat anti-mouse IgG (Molecular Probes). Images were acquired using a fluorescence microscope (Nikon Eclipse 80i). Full length cDNA for Pdzd2 with N-terminus myc-tag in pCIneo (Promega) was transfected into cultured CHO-SNS22 cells using lipofectamine 2000 as described (Okuse et al., 2002).

### GST pull-down

GST pull-down assay was performed as described (Poon et al., 2004). The cDNA for haemagglutinin (HA)-tagged rat Pdzd2 C-terminal fragment (amino acid positions (aa) 2567–2767) was cloned into NcoI/XbaI sites of pBS500 vector in order to produce green fluorescent protein (GFP)-fused Pdzd2 C-terminal polypeptide whose estimated molecular weight is 60 kDa. The cDNA coding intracellular loop between domains 2 and 3 of rat Na_V_1.8 (aa 893 to 1148) and Na_V_1.7 (aa 968 to 1157) were amplified by RT-PCR from rat DRG mRNA. The amplified cDNA fragments were sequenced, and subcloned into pGEX-5X-1 vector in order to generate glutathione S-transferase (GST)-fused intracellular loops of Na_V_1.8 and Na_V_1.7. The GST fusion proteins were expressed in BL21 cells and purified using glutathione sepharose beads.

### Electrophysiology

Wistar rats (> P21) were killed in accordance with home office guidelines and their DRG removed and dissociated using collagenase type XI and a bacterial protease (Sigma), according to the method of Baker and Bostock (1997). Primary cultures of DRG neurons were grown on poly-l-lysine coated coverslips and maintained in a 5% CO_2_ incubator. DNA microinjection into the nuclei of cultured DRG neurons was performed as described (Okuse et al., 2002). Fluorescently labelled siRNA was transfected into the neurons using oligofectamine 24 h after plating-out the cultures. The following siRNA duplexes were used; negative control siRNA, UUCUCCGAACGUGUCACGUdTdT; Pdzd2-specific siRNA, GGCAAGGGCCUUGGCUUUAdTdT (Qiagen). Microinjected or transfected neurons only were studied. Whole-cell voltage-clamp recordings from small (< 25 μm apparent diameter) primary sensory neurons in culture were made at 2 and 3 days following siRNA transfection. An Axopatch 200B amplifier (Axon Instruments, Union City, California, USA) was used to record membrane currents in response to families of depolarizing clamp-steps. Pulse protocols (Pclamp 9, Axon Instruments) were generated by a PC and recordings made on-line, filtered at 5 kHz (4-pole Bessel filter) and sampled at 20 kHz. An estimate of cell capacitance was obtained using the capacity transient cancellation procedure provided by the amplifier, while simultaneously generating an estimate of the series-resistance. Electrodes were made from thin-walled glass (Harvard Apparatus, Edenbridge, Kent, UK) and had an initial resistance of between 1.5 and 2 MΩ when initially filled with intracellular solution. During recording, series-resistance compensation was set near 70% with a nominal feed-back lag of 12 μs. Using a holding potential of − 80 mV, and a 20 ms pre-pulse, Na currents were elicited by incremental depolarizing clamp-steps. Current recordings were usually the average of 3 responses to the repeated voltage-clamp protocol. Because Na_V_1.9 (commonly referred to as the persistent Na^+^ current) optimally requires a more negative holding potential, and internal F^−^ ions eventually lead to its run-down (Baker et al., 2003), the TTX-r currents recorded were attributable to Na_V_1.8. Additionally, the voltage dependence and kinetics of the residual TTX-r current recorded were consistent with it being generated by Na_V_1.8. Post-hoc analysis included dividing the peak current values by the estimated value of membrane capacitance, allowing an estimate of peak current density to be made for each neuron. Measurements of TTX-r current density were averaged and expressed as means ± s.e.m. All recordings were made at room temperature. The presence of 250 nM TTX in the extracellular solution was used to eliminate TTX-s Na^+^ currents, in a manner identical to that used by Okuse et al. (2002). The TTX-r Na^+^ currents attributable to Na_V_1.8 were further isolated by using blockers of both K^+^ and Ca^2+^ currents in the recording solutions. The extracellular solution contained (in mM): NaCl 43.3, Tetraethylammonium Chloride 96.7, HEPES 10, CaCl_2_ 2.1, MgCl_2_ 2.12, 4-Aminopyridine 0.5, CsCl 10, KCl 7.5, and CdCl_2_ 0.1. The intracellular solution contained (in mM): CsCl 130, CsF 13, EGTA (Na) 3, Tetraethylammonium Chloride 10, HEPES 10, CaCl_2_ 1.21, ATP(Mg) 3 mM, and GTP (Li) 500 μM. With the addition of CsOH, all solutions were buffered to pH 7.2–7.3. With the exception of TTX, reagents were purchased from Sigma-Aldrich (Poole, Dorset, UK). TTX was obtained from Alomone Labs (TCS Biologicals, Botolph Claydon, Bucks, UK).

### Behavioural studies of Pdzd2 deficient mice

Pdzd2 deficient mice (K.M. Yao and K.S.E. Cheah, in preparation) were generated by insertion of the RASAFRAY viral vector (Chen et al., 2004). A complete shutdown of expression of Pdzd2 variants 1, 2 and 5 in the Pdzd2 deficient mice were confirmed by RT-PCR. All behavioural studies were performed on animals aged between 8 and 16 weeks as described (Foulkes et al., 2006).

### Quantitative real-time RT-PCR

Mouse DRG total RNA was extracted using RNeasy kit (Qiagen). 0.5 micrograms of total RNA were reverse transcribed using cDNA synthesis kit (Invitrogen). 0.5 micrograms of each RNA sample was incubated similarly in the absence of reverse transcriptase to ensure that PCR products resulted from amplification of specific mRNA rather than from genomic DNA contamination. The expression of p11mRNA was measured by quantitative real-time RT-PCR using the MX3005P QPCR system (Stratagene). p11 primers (forward 5′-ACGCCATGGAAACCATGATG-3′; reverse 5′-GCTCTGGAAGCCCACTTTGC-3′) were used. Primers for the amplification of the housekeeping gene cyclophilin-A (forward 5′-TATCTGCACTGCCAAGACTGAGTG-3′; reverse 5′-CTTCTTGCTGGTCTTGCCATTCC-3′) were used to normalise the amount of cDNA present in each reaction. qRT-PCR reaction was performed in 20 μl reactions, containing 5 μl cDNA template, 1 μl of each forward and reverse primer (1 μM), 10 μl Brilliant SYBR Green QPCR Master Mix (Stratagene) and 3 μl of nuclease-free water. The thermal profile used to amplify the PCR products included an initial 10 min incubation at 95 °C, followed by 40 cycles of; denaturation at 95 °C for 30 s, annealing at 55 °C for 1 min, and elongation at 72 °C for 30 s. The fluorescence readings were recorded after each 72 °C step. Dissociation curves were performed after each PCR run to ensure that a single PCR product had been amplified per primer set. Each sample was measured in triplicate. The p11mRNA levels of each animal were normalised to the cyclophilin-A. For quantitative purposes, standard calibration curves generated from a series of dilutions containing 10^8^, 10^7^, 10^6^, 10^5^, 10^4^, 10^3^, 10^2^ copies of p11 cDNA were prepared, and run in the same condition as mentioned above. The PCR efficiencies for p11 primer pairs were above 94%. Changes in gene expression levels of p11mRNA were quantitated by calculating the absolute copy number for each sample based on the standard curve.
